# Inhibition of heat shock protein 90 improves pulmonary arteriole remodeling in pulmonary arterial hypertension

**DOI:** 10.18632/oncotarget.10855

**Published:** 2016-07-26

**Authors:** Guo-Kun Wang, Song-Hua Li, Zhi-Min Zhao, Su-Xuan Liu, Guan-Xin Zhang, Fan Yang, Yang Wang, Feng Wu, Xian-Xian Zhao, Zhi-Yun Xu

**Affiliations:** ^1^ Institution of Cardiac Surgery, Department of Cardiovascular Surgery, Changhai Hospital, Second Military Medical University, Shanghai, China; ^2^ Department of Cardiology, Changhai Hospital, Second Military Medical University, Shanghai, China; ^3^ Department of Cardiology, 98th Military Hospital, Huzhou, Zhejiang, China

**Keywords:** pulmonary arterial hypertension, heat shock protein 90, pulmonary artery smooth muscle cells, pulmonary arteriole remodeling, Pathology Section

## Abstract

While the molecular chaperone heat shock protein 90 (HSP90) is involved in a multitude of physiological and pathological processes, its role relating to pulmonary arterial hypertension (PAH) remains unclear. In the present study, we investigated the effect in which HSP90 improves pulmonary arteriole remodeling, and explored the therapeutic utility of targeting HSP90 as therapeutic drug for PAH. By Elisa and immunohistochemistry, HSP90 was found to be increased in both plasma and membrane walls of pulmonary arterioles from PAH patients. Moreover, plasma HSP90 levels positively correlated with mean pulmonary arterial pressure and C-reactive protein. In a monocrotaline-induced rat model of PH, we found that 17-AAG, a HSP90-inhibitor, alleviated the progress of PH, demonstrated by lower pulmonary arterial pressure and absence of right ventricular hypertrophy. Immunohistochemical staining demonstrated that 17-AAG improved pulmonary arteriole remodeling on the basis of reduced wall thickness and wall area. The inflammatory response attributed to PH could be attenuated by 17-AAG through reduction of NF-κB signaling. Moreover, 17-AAG was found to suppress PDGF-stimulated proliferation and migration of pulmonary artery smooth muscle cells (PASMCs) through induction of cell cycle arrest in the G1 phase. In conclusion, HSP90 inhibitor 17-AAG could improve pulmonary arteriole remodeling via inhibiting the excessive proliferation of PASMCs, and inhibition of HSP90 may represent a therapeutic avenue for the treatment of PAH.

## INTRODUCTION

Pulmonary arterial hypertension (PAH) is clinically characterized by pulmonary arteriole contraction resulting in chronic elevation of pulmonary vascular resistance, right ventricular remodeling, dysfunction and ultimately failure [1, 2]. While the pathogenesis of PAH is not fully characterized, pulmonary vasoconstriction, vascular remodeling and *in-situ* thrombosis formation are believed to represent central components of the pathobiology. It has been reported that the extensive proliferation of pulmonary artery smooth muscle cells (PASMCs) in the medial layer of pulmonary arteries may be among the most prominent feature of PAH [3, 4].

Heat shock protein 90 (HSP90), an ubiquitous chaperones, is involved in numerous physiological and pathological processes, especially in cancers [5]. HSP90 plays important roles in cell division by regulating maturation of signaling proteins including: kinases, steroid hormone receptors and key oncogenic proteins [6–8]. Recently, HSP90 inhibitor, 17-allylamino-17-demethoxygeldamycin (17-AAG), was demonstrated to attenuate formation of atherosclerotic plaques by reducing inflammatory responses and suppress migration and proliferation of vascular smooth muscle cells [9, 10]. Herein, we detected the expression of HSP90 in CHD patients with PAH, investigated the impact of 17-AAG on pulmonary arteriole remodeling and PASMCs growth, and explored the potential utility of HSP90 inhibitors as therapeutic avenue for PAH.

## RESULTS

### Patients characteristics

Plasma from 27 CHD patients with PAH (3 patients with atrial septal defects, 15 patients with ventricular septal defects, 6 patients with patent ductus arteriosus, 2 patients with coronary artery fistula, 1 patient with double-outlet right ventricle with mitral atresia), 39 CHD patients without PAH (all with left-to-right shunting), and 23 healthy control, were analyzed. Demographic, laboratory, clinical, and hemodynamic data are summarized in Table 1. Invasive hemodynamics was not obtained in the control group. The majority of patients were WHO functional Class I or Class II, and had not received any pharmacological treatment. There were no significant differences in the age or gender composition of the three groups (*P* > 0.05).

**Table 1:** Clinical characteristics of patients and healthy volunteers enrolled in this study

Variable	PAH (*n* = 27)	CHD (*n* = 39)	Healthy (*n* = 23)	*P*
Female [n (%)]	18 (66.7)	22 (56.4)	13 (56.5)	0.665
Age [year]	43.8±12.3	37.4±15.5	38.7±8.5	0.142
BMI [kg/m^2^]	22.2±3.6	20.8±3.4	22.3±2.3	0.110
TC [mmol/L]	4.42±1.02	4.38±0.73	4.00±0.86	0.166
TG [mmol/L]	1.19±0.70	1.17±0.61	0.84±0.35	0.065
LDL [mmol/L]	2.59±0.77	2.65±0.56	2.82±0.21	0.330
WBC [×10^9^/L]	6.03±1.43	6.32±1.62	6.02±1.12	0.632
NEUT [%]	61.96±10.17	61.43±9.02	62.8±5.61	0.845
WHO functional class Class I [n(%)] Class II [n(%)] Class III [n(%)] Class IV [n(%)]	20 (74.1%) 4 (14.8%) 2 (7.4%) 1 (3.7%)	34 (87.1%) 4 (10.3%) 1 (2.6%) 0 (0.0)	-	0.469
dPAP [mmHg]	53.6±20.9	28.2±4.6	-	<0.01
sPAP [mmHg]	23.96±13.1	9.87±3.0	-	<0.01
mPAP [mmHg]	36.6±13.7	16.7±2.3	-	<0.01
Pro-BNP [pg/mL]	560.1±1148.1	63.6±102.7	-	<0.01
CRP [mg/L]	3.78±4.62	0.57±0.72	-	<0.01
IL6 [pg/mL]	8.11±22.7	1.85±0.74	-	<0.01

BMI, body mass index; TC, total cholesterol; TG, total glyceride; LDL, low-density lipoprotein; WBC, white blood cell; NEUT, neutrophil; dPAP, diastolic pulmonary artery pressure, sPAP, systolic pulmonary artery pressure; mPAP, mean pulmonary artery pressure; pro-BNP, pro-brain natriuretic peptide; CRP, C reaction protein; IL6, interleukin-6. The quantitative data were expressed as mean ± standard deviation (SD).

### Elevation of HSP90 levels in plasma and lung tissue of patients with PAH

**Figure 1:**
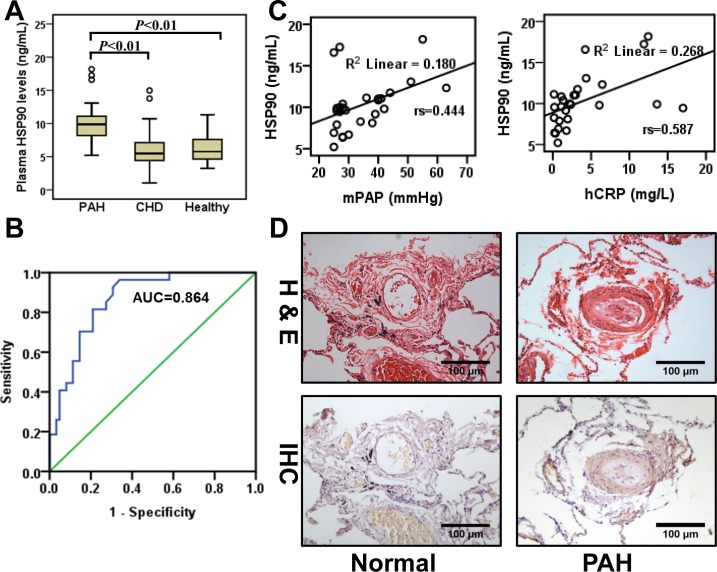
HSP90α expression in plasma and lung tissue from PAH patients **A.**, Detection of HSP90 in plasma from CHD patients with PAH (PAH, *n* = 27), CHD patients without PAH (CHD, *n* = 39), and healthy volunteers (Healthy, *n* = 23). **B.**, Receiver Operating Characteristic curves were applied to evaluate the sensitivity and specificity of HSP90α on all donors (*n* = 89). The area under the ROC curve of HSP90α was 0.864 (sensitivity 92.6%, specificity 69.4%). **C.**, Spearman correlation test was applied to analyze the correction between HSP90α and clinical parameters on CHD patients with PAH (*n* = 27). **D.**, Representative images of hematoxylin-eosin staining (H&E) and immunohistochemistry (IHC) of HSP90α staining in lung tissues lesions. Lung tissue specimens were collected from CHD patients with PAH (*n* = 11) or from autopsy (*n* = 3).

It has been previously demonstrated that the level of plasma Hsp90α positively correlates with tumor malignancy [11]. Therefore, we evaluated HSP90α expression in plasma from the three groups. Plasma HSP90α levels were significantly higher in patients with PAH as compared to CHD-only and control groups (11.36±5.67 ng/mL *vs* 6.24±1.99 ng/mL and 6.17±2.98 ng/mL, *P* < 0.05). There was no difference between CHD and control groups (Figure 1A). Receiver Operating Characteristic (ROC), applied on data from all donors, demonstrated that HSP90α reflected strong separation between PAH and non-PAH groups, with an area under curve (AUC) of 0.864 (95% confidence interval 0.790-0.939, Figure 1B). Correlation analysis between HSP90α plasma content and clinical parameters was performed on the PAH group. Spearman correlation test demonstrated that plasma HSP90α level positively correlated with mPAP (r_s_ = 0.444, *P* < 0.05) and CRP (r_s_ = 0.587, *P* < 0.01). No associations with other clinical parameters were observed (Figure 1C and Table S1). We also collected lung tissue from CHD patients with PAH (*n* = 11) and normal lung tissue from autopsy (*n* = 3). In CHD patients with PAH, a thicker wall and cell proliferative medial membrane were observed by hematoxylin-eosin staining in the pulmonary arteriole. Immunohistochemistry analysis detected HSP90 expression in pulmonary arteriole in 81.8% (9/11) CHD patients with PAH, while no expression was observed in normal lung tissue (Figure 1D). These results suggest that HSP90 might be involved in the progress of PAH.

### Inhibition of HSP90 may alleviate the progress of MCT-induced PH

**Figure 2:**
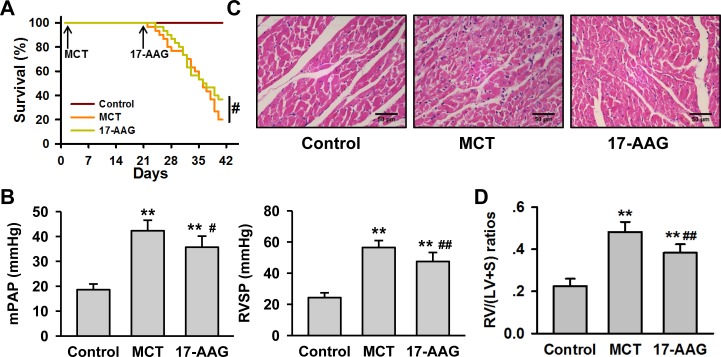
HSP90-inhibition alleviates MCT-induced PH **A.**, Survival curves of MCT-induced rats with or without 17-AAG treatment (*n* = 30 initially for each group). #*P* < 0.05. **B.**, Right heart catheterization analysis of mPAP and RVSP on the surviving rats (*n* = 6 in MCT group, and *n* = 11 in 17-AAG group). **P < 0.01 *vs* control group, and #*P* < 0.05, ##*P* < 0.01 *vs* MCT group. **C.**, Representative images of hematoxylin-eosin staining in heart tissues lesions of the survived rats (*n* = 6 in MCT group, and *n* = 11 in 17-AAG group). **D.**, Analysis of right ventricle hypertrophy index (RVHI) of the surviving rats (*n* = 6 in MCT group, and *n* = 11 in 17-AAG group). **P < 0.01 *vs* control group, and ##*P* < 0.01 *vs* MCT group.

Previous reports have demonstrated that HSP90 is decreased in lung tissue from PH rats by western blot analysis [12, 13]. Interestingly, immunohistochemistry assay highlighted cell-type specific staining in which HSP90 was significantly increased in membrane walls of pulmonary arterioles from MCT group in spite of the overall reduction in the lung tissue (Figure S1). To test whether inhibition of HSP90 impacted the development of PH, we evaluated the effect of 17-AAG using a monocrotaline (MCT)-induced PH rat model. While no animals died during the modeling stage (3 weeks after MCT injection), in the treatment stage (3 weeks after 17-AAG injection) 80% of animals (24/30) died of heart failure in the MCT group, while only 63.3% of animals (19/30) died in the 17-AAG group (Figure 2A). To understand the cause of these findings, right heart catheterization was performed on the surviving rats from each group. Compared with controls, rats in MCT group exhibited significant increase in mPAP (Control group: 18.63±2.29 mmHg; MCT group: 42.30±4.20 mmHg, *P* < 0.01) and RVSP (Control group: 24.3±3.0 mmHg; MCT group: 56.5±4.0 mmHg, *P* < 0.01). Treatment with 17-AAG significantly reduced the mPAP (35.70±4.43 mmHg, *P* < 0.05 *vs* MCT group) and RVSP (47.5±5.8 mmHg, *P* < 0.05 *vs* MCT group, Figure 2B). Histological staining of right ventricle in the MCT group revealed disorganized myocardial cells as compared to the control group. Notably, 17-AAG treatment effectively alleviated these structural changes (Figure 2C). The right ventricle hypertrophy index in MCT group was significantly increased compared with control group (0.482±0.05 *vs* 0.230±0.03, *P* < 0.01), but apparently fell in the 17-AAG-treated group (0.383±0.04, *P* < 0.05 *vs* MCT group, Figure 2D).

### Inhibition of HSP90 improved the remodeling of pulmonary arteriole in PH

**Figure 3:**
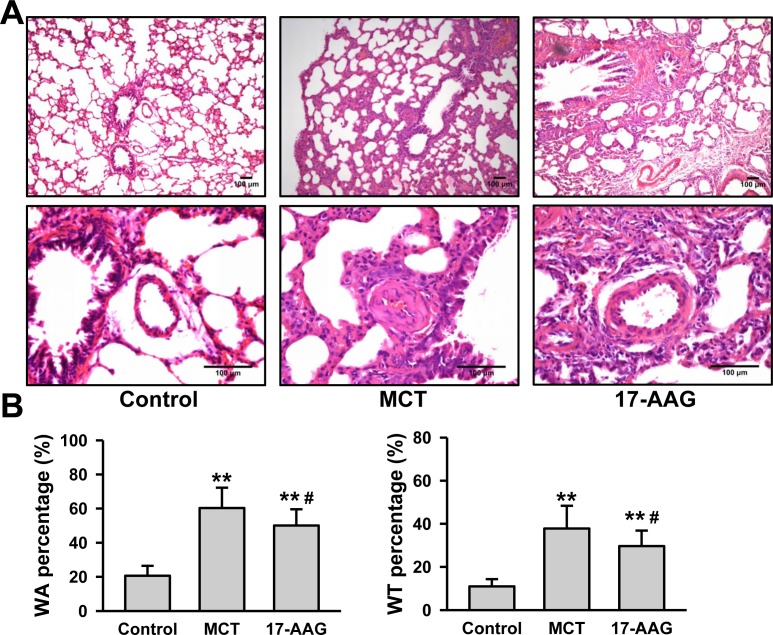
HSP90-inhibition improves pulmonary arteriole remodeling in PH **A.**, Representative images of hematoxylin-eosin staining in lung tissues lesions of the surviving rats (*n* = 6 in MCT group, and *n* = 11 in 17-AAG group). **B.**, Analysis of wall area (WA) and wall thickness (WT) in rats that survived until the end of a 6-week study period (*n* = 6 in MCT group, and *n* = 11 in 17-AAG group). Ten pulmonary arterioles were randomlystructural integrity from hematoxylin-eosin staining images of lung tissues lesions by. **P < 0.01 *vs* control group, and #*P* < 0.05 *vs* MCT group.

Pulmonary arteriole remodeling is an important element of the pathogenesis of PH. Therefore, microscopic analysis of structure characteristic was performed on paraffin sections of rat lung tissues after 6 weeks of MCT injection. The pulmonary arterial walls in control group presented clearly with high integrity and normal arterial tunica media thickness. However, thickening and swelling of the pulmonary artery's tunica media and alveolar septum, stenosis of the arterial lumen could be observed in MCT group. Treatment with 17-AAG significantly alleviated these changes (Figure 3A). Image J software was used to measure the parameter of the pulmonary arterioles, including the internal/external diameter and the area of vessel/lumen (Table S2), to calculate the wall thickness percentage (WT%) and wall area percentage (WA%). When compared against the control group (WT%: 10.97±3.34%, WA%: 20.61±5.85%), there were remarkable increase on WT% (37.81±10.56%) and WA% (60.30±11.94%) in the MCT group (*P* < 0.01 respectively). However, 17-AAG treatment markedly reduced the increase in both WT% (26.16±13.59%) and WA% (50.07±9.55%) (*P* < 0.01 *vs* MCT group respectively), although still remained significantly elevated compared with those of the control group (*P* < 0.05 respectively, Figure 3B).

### Inhibition of HSP90 decreased inflammatory signaling pathways in PH

**Figure 4:**
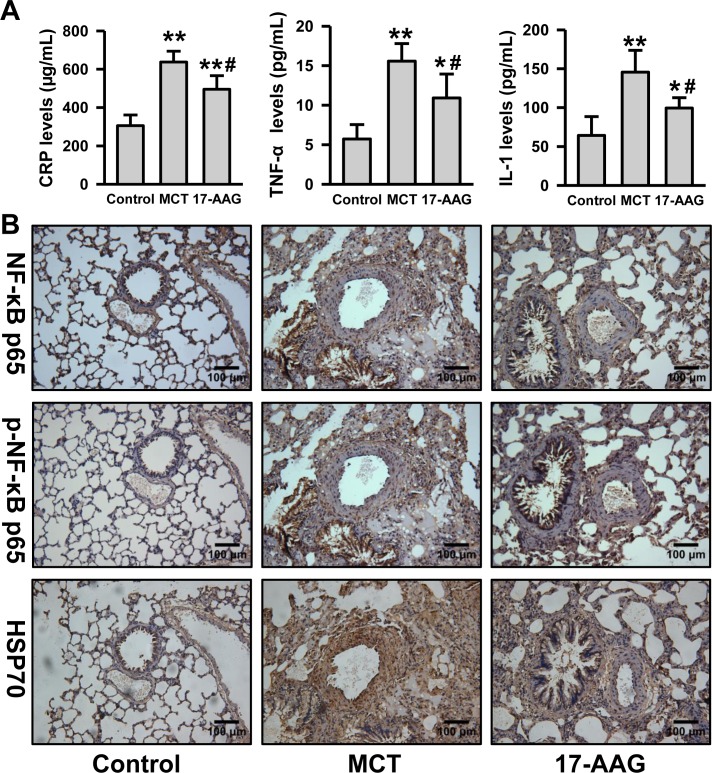
Inhibition of HSP90 decreased inflammatory and NF-κB signaling in PH **A.**, Analysis of the inflammatory cytokine (CRP, TNF-α and IL-1) levels in plasma in rats (*n* = 6 in MCT group, and *n* = 11 in 17-AAG group). **P < 0.01 *vs* control group, and #*P* < 0.05, ##*P* < 0.01 *vs* MCT group. **B.**, Representative images of immunohistochemistry of p65, phosphorylated p65 and HSP70 in lung tissues lesions (*n* = 6 in MCT group, and *n* = 11 in 17-AAG group).

Considering the positive correlation between HSP90α and CRP plasma levels in CHD patients with PH, we detected the plasma inflammatory cytokine levels in rats. Compared with the control group, plasma CRP, TNF-α, and IL-1 levels were notably increased in the MCT group (*P* < 0.01), while the levels approached baseline in rats treated with 17-AAG (*P* < 0.05 *vs* MCT group respectively, Figure 4A). We evaluated NF-κB signaling and the expression of anti-inflammatory HSP70 on rat lung tissues by immunohistochemistry. When compared with the MCT group, 17-AAG-treated rats showed weaker staining for NF-κB p65, and weaker nuclear staining for phosphorylated NF-κB p65. It is confused that the staining for HSP70 was also detected weaker in 17-AAG-treated group (Figure 4B and Table S3). Nevertheless, these results suggest that 17-AAG might suppress inflammation by decreasing NF-κB signaling and upregulating of HSP70 expression in PAH.

### Inhibition of HSP90 prevented PASMCs proliferation and migration induced by PDGF-bb

**Figure 5:**
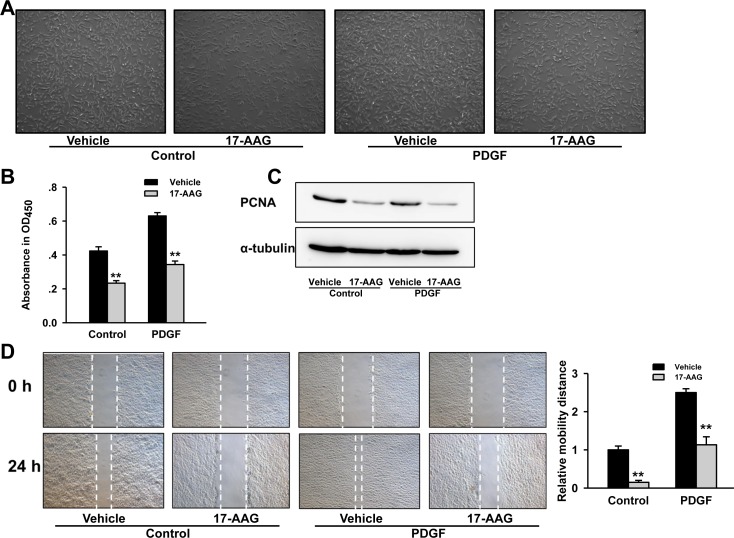
Inhibition of HSP90 reduced PASMCs proliferation and migration induced by PDGF-bb **A.**, Microscopic observation of PASMCs population after 17-AAG treatment. **B.**, Affect of 17-AAG on proliferation of PASMCs with or without PDGF-bb stimulation. Data represent the mean of three independent experiments ±Standard deviation. **C.**, Western blot analysis of proliferating cell nuclear antigen (PCNA) expression in PASMCs after 17-AAG treatment. α-Tubulin was used as an internal control. **D.**, Affect of 17-AAG on migration of PASMCs with or without PDGF-bb stimulation. Data represent the mean of three independent experiments ±Standard deviation.

Since excessive proliferation and migration of PASMCs is a central pathologic feature of pulmonary vascular remodeling in PH, we investigated the effect of HSP90 inhibitor on PASMCs growth. Microscope observation showed that PASMCs growth were significantly inhibited in 17-AAG treated groups (Figure 5A). CCK-8 cell viability assay demonstrated an increase of ~1.6-fold following 24-hours of PDGF-bb stimulation, whereas 17-AAG caused a reduction irrespective of stimulation (*P* < 0.01, Figure 5B). Western blot assay also indicated that protein expressions of proliferating cell nuclear antigen (PCNA) were remarkably reduced by 17-AAG both in control and PDGF-stimulated groups (Figure 5C). However, flow cytometry assay was unable to detect any significant change in apoptosis induced by 17-AAG (Figure S2). Furthermore, 17-AAG-treatment displayed a strong inhibitory effect on PASMCs migration (Figure 5D). Taken together, these results suggest that HSP90 inhibition can suppress proliferation and migration of PASMCs, thereby improving pulmonary arteriole remodeling of PAH.

### Inhibition of HSP90 induced cell cycle arrest in G1 phase *via* decreasing CDK4 and CCND1 expression

**Figure 6:**
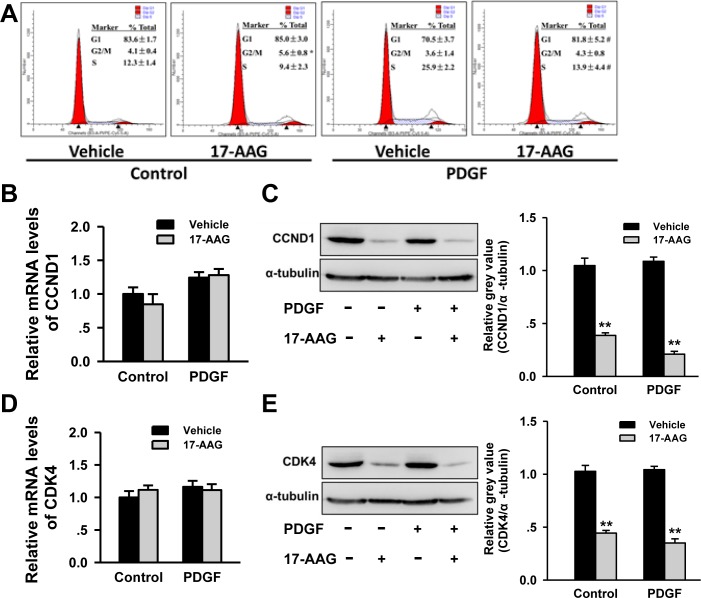
Inhibition of HSP90 induced cell cycle arrest by regulation of cell cycle regulatory proteins stability **A.**, Affect of 17-AAG on cell cycle of PASMCs with or without PDGF-bb stimulation. Data represent the mean of three independent experiments ±Standard deviation. **B.** and **C.**, Real-time PCR and western blot analysis of CCND1 in PASMCs after 17-AAG treatment. Data represent the mean of three independent experiments ±Standard deviation. **P < 0.01 *vs* vehicle. **D.** and **E.**, Real-time PCR and western blot analysis of CDK4 in PASMCs after 17-AAG treatment. Data represent the mean of three independent experiments ±Standard deviation. **P < 0.01 *vs* vehicle.

HSP90 had been reported to impact the cell cycle of VSMCs in atherosclerosis progression, and as such we proceeded to investigate the impact of 17-AAG-treatment on cell cycle progression in PASMCs. Flow cytometry analysis identified an increasing G1 phase-cell population (70.5±3.5% to 81.8±5.2%, *P* < 0.05) and decreased the S phase-cell population (25.9±2.2% to 13.9±4.4%, *P* < 0.05) in 17-AAG-treated cells in a PDGF-bb-stimulated environment. No significant changes were induced by 17-AAG in the basal group (Figure 6A). Progression of the cell cycle is strictly controlled by cyclins and cyclin-dependent kinases, including cyclin-dependent kinase 4 (CDK4), which may be affected by HSP90 and the co-chaperone adaptor CDC37. As such, we assessed expression changes of these genes by real-time PCR and western blot analysis. While no significant changes in mRNA levels were observed, CDK4 protein levels significantly decreased after 17-AAG treatment both in basal and PDGF groups (Figure 6B and 6C). Correspondingly, the protein levels of CCND1 were also significantly decreased in the 17-AAG-treated groups, but similarly mRNA levels were unaffected (Figure 6D and 6E). CDK4 co-chaperone adaptor CDC37 protein expression was unchanged by 17-AAG treatment (Figure S3). These results demonstrate that HSP90-inhibition induced cell cycle arrest by modulating the stability of cell cycle regulatory proteins.

## DISCUSSION

It is well established that heat shock proteins are involved in regulation of many signaling pathways, which are crucial to normal development and the pathobiology of numerous disease states. As such, application of a HSP90 inhibitors as therapeutic drugs for PAH treatment, might affect other pathological and physiological processes, such as oxidative stress or neoangiogenesis [14, 15]. Further studies are required to clarify the value of HSP90 inhibitor as a treatment avenue for PAH. Although clinical trials in cancer patients had indicated that the combination of HSP90 inhibitors with other drugs display acceptable toxicity levels [16, 17], our results showed that 17-AAG caused a remarkable reduction on PASMCs growth in normal condition, and the PCNA expression was decreased by 17-AAG in a control setting. The emergence of more potent and water soluble derivative, such as 17-DMAG or IPI-504, might improve the utility of HSP90 inhibitors as therapeutic drug.

Given that HSP90 can be found in the extracellular environment, the plasma HSP90α level positively correlated with tumor malignancy and metastasis [11, 18]. Our results demonstrated that plasma HSP90αlevels were elevated compared with those from CHD patients without PAH and healthy controls. Moreover, HSP90α positively correlated with mPAP and CRP. ROC analysis further indicated that HSP90α might be a potential biomarker for PAH diagnosis. It should be noted that plasma HSP90α levels in PAH patients were still far less than those levels observed in cancer patients.

The inflammatory response plays an important role in the development of PAH [19]. Our result from clinical and animal experiments showed that 17-AAG might reduce the inflammatory response in PAH by inhibiting HSP90 activity. Although it was reported that immunosuppression was invalid for PAH, our results suggest that early anti-inflammatory treatment might be effective for improvement of pulmonary arterioles remodeling. However, whether the pulmonary benefit observed after 17-AAG treatment was related to a local or systemic effect remained unclear. Based on the contribution of HSP90 to inflammation, the effect of 17-AAG treatment should be the systemic effect for the pulmonary benefit.

The present work has led us to conclude that HSP90-inhibition may represent an effective treatment avenue for PAH therapy. Currently, HSP90 inhibitor therapy is plagued by numerous challenges relating to bioavailability, toxicity and stability. Moreover, the molecular mechanisms and the drug sensitivity genes have not been entirely elucidated. Development of more efficient and specific HSP90 inhibitors would promise new therapy strategy for many diseases in future.

## MATERIALS AND METHODS

### Study populations

All PAH patients enrolled in this study received treatment for CHD at Department of Cardiovascular Surgery or Department of Cardiology in Changhai Hospital between July 2013 and February 2014. PAH diagnoses was made following right heart catheterization, and an evaluation of the diagnostic criteria as reported by the 5th World Symposium in France in 2013 [2]. Patients with cancer, infection, fever, trauma or stress were excluded from this study. Age- and sex-matched CHD patients without PAH (mPAP < 18 mmHg), and healthy volunteers without history of cardiovascular disease were recruited as a control group.

This study was carried out in accordance to the principles of the Declaration of Helsinki and approved by the Medical Ethics Committee in Shanghai Changhai Hospital. Written informed consent was obtained from all the participants prior to enrollment.

### Animal model of PH

Ninety male Sprague-Dawley rats, weighing from 200 to 250 g, were randomly divided into three groups: Control, MCT and 17-AAG groups. PH was induced by a single subcutaneous injection of monocrotaline (MCT, 50 mg/kg, Sigma), while control rats were injected with normal saline. In the 17-AAG group, rats were subcutaneous injection of 17-AAG (30 mg/kg, Sigma) every 2 days for 21 days after MCT injection. Similar treatment schedules were used for mock (saline)-treated control and MCT groups.

The animal work performed in this study was approved by institutional review board of the local university, and the experiment protocols were carried out according to the guidelines for the care and use of laboratory animals established by the US National Institutes of Health.

### Right heart catheterization

Rats were anesthetized intraperitoneally with chloral hydrate (300 mg/kg). A PE-50 silicone catheter (0.9 mm outer diameter) was introduced into the right jugular vein, passing through right atrium, the tricuspid valve and right ventricle, and into the pulmonary artery. A multichannel physiologic recorder (BIOPAC) was connected to the other end of the catheter. The position of the catheter was determined by the typical wave form of the pressure. The systolic pulmonary artery pressure (sPAP) and diastolic pulmonary artery pressure (dPAP) were recorded simultaneously.

### Right ventricular hypertrophy index (RVHI)

After measuring pulmonary vascular hemodynamics, the rats were sacrificed by CO_2_ inhalation followed by cervical dislocation. Tissues and organ (heart, lung, liver, and kidney) were isolated. The right ventricle (RV) wall was dissected from the left ventricle (LV) and ventricular septum (S). The wet weight of the RV and LV+S was determined respectively, and RVHI was calculated as the weight ratio of RV/(LV+S).

### Wall thickness (WT) and wall area (WA)

The left lungs were embedded in paraffin after fixation by 4% paraformaldehyde, and the sections

### Immunohistochemistry

The deparaffinized and rehydrated sections were incubated in warm citric acid/sodium citrate buffer (pH 6.0) for antigen retrieval. After blocking the endogenous peroxidases, the sections were incubated with the primary antibodies overnight followed by incubation with HRP-conjugated secondary antibody. Negative controls using the corresponding IgG for checking non-specific staining.

### Cell culture and intervention

Male Sprague-Dawley rats were sacrificed by CO_2_ inhalation followed by cervical dislocation. Pulmonary artery smooth muscle cells (PASMCs) were obtained by collagenase digestion of pulmonary artery and cultured in Dulbecco modified Eagle medium supplemented with 10% fetal bovine serum. PASMCs (passage 4-6) were seeded in plates, and stimulated by PDGF-bb (10 ng/mL) with or without 17-AAG (1 μmol/L). Cells were harvested after 24 hours for further observation and analysis.

### Flow cytometry for cell cycle and apoptosis

For cell cycle analysis, PASMCs were fixed with cold 70% ethanol, and incubated in staining solution (20 μg/mL propidium iodide and 50 μg/mL RNase A in phosphate-buffered saline) at room temperature for 30 minutes. For detection of apoptosis, PASMCs were harvested and stained by Annexin V/propidium iodide at room temperature for 15 minutes. All samples were analyzed using FACSort flow cytometer and Cell Quest Pro (BD).

### Real-time PCR

Total RNA (500 ng) extracted from PASMCs by trizol reagent according to the protocol of the manufacturer (life technologies). The cDNA was generated by using PrimeScript RT reagent Kit (TAKARA) with oligo-dT and random primer. Real-time PCR was performed on a LightCycler 480 II quantitative PCR system (Roche) using SYBR Green. Primers used in the amplification reaction were in Table S3.

### Western blot

Protein was extracted from PASMCs by RIPA buffer plus protease inhibitors. Normalized amounts of protein (about 30 μg) was separated by SDS-PAGE and transferred to the PVDF membrane. After blocking by 5% non-fat milk in phosphate-buffered saline with 0.1% Tween 20, the membrane was incubated with diluted primary antibodies overnight followed by incubation with HRP-conjugated secondary antibody. Proteins were visualized by ECL Plus Western Blotting Substrate (Thermo Scientific) on ImageQuant LAS500 (GE). α-tubulin was detected as loading control.

### Statistical analysis

All statistical analyses were performed by SPSS version 17.0. The qualitative data was compared with Fisher's exact test. The quantitative data was first evaluated whether they followed the normal distribution by the Shapiro-Wilk test. The data of non-normal distribution was performed by Kruskal-Wallis test. The data of normal distribution was further performed by Levene's test for homogeneity of variance analyses. One-way ANOVA was performed to assess the homogeneity of variance, and Kruskal-Wallis test was performed on the data that did not meet the homogeneity of variance. Relationship between levels of HSP90 and other clinical parameters were analyzed by Spearman test. Receiver operating characteristic (ROC) curves were established for discriminating PAH patients from the ones with CHD. All *P*-values are two-sided and less than 0.05 was considered statistically significant difference.

## SUPPLEMENTARY MATERIALS


