# Supplementary material for: Inhibition of heat shock protein 90 improves pulmonary arteriole remodeling in pulmonary arterial hypertension
Source: Oncotarget. 2016 Jul 26;7(34):54263–73. doi: 10.18632/oncotarget.10855 (PMC5342340; doi:10.18632/oncotarget.10855)
Supplement: Supplementary file 1 [file oncotarget-07-54263-s001.pdf]

# Inhibition of heat shock protein 90 improves pulmonary arteriole remodeling in pulmonary arterial hypertension

**Supplementary Material**

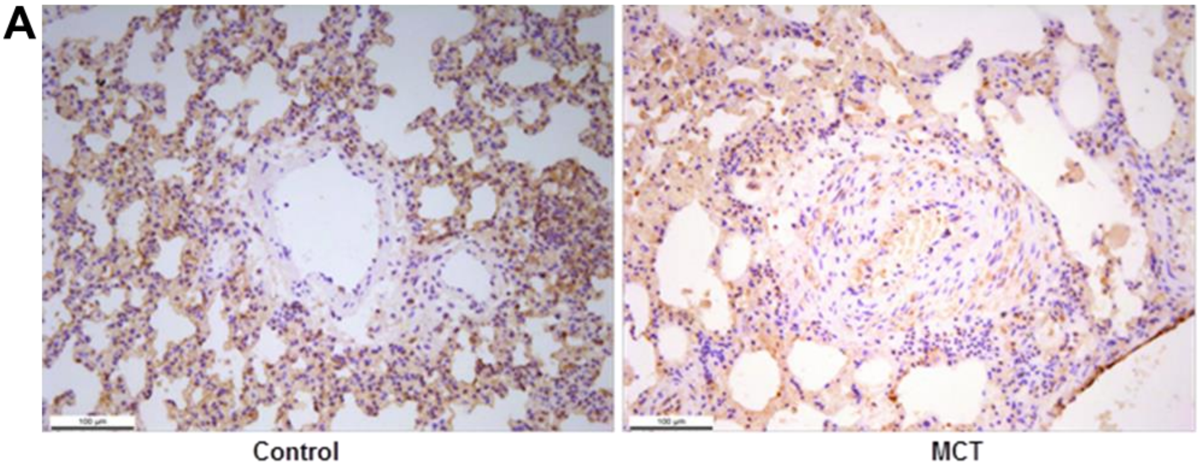


B

| | Control (n=6) | MCT (n=6) |
|---|---|---|
| HSP90α immunointensity | 0.29±0.46 | 1.38±0.49(**) |
| % HSP90α positive | 3.18±1.70 | 13.0±3.48(**) |

Figure S1: Immunohistochemistry detection of HSP90α in pulmonary arterioles of rats lung tissues lesions. A, Representative immunohistochemistry images of HSP90α staining in pulmonary arterioles of rats lung tissues lesions (n=6). Compared with control group, HSP90 was significantly increased in membrane walls of pulmonary arterioles from MCT group in spite of the overall reduction in the lung tissue. B, HSP90α expression was estimated by immunostaining intensity and percentage of HSP90α positive cells in pulmonary arterioles. Immunostaining intensity was rated as follows: 0, none; 1, weak; 2, moderate; and 3, intense. Percentage of HSP90α positive cells was calculated by HSP90α-positive pulmonary arterioles cells. The quantitative data were expressed as mean ± standard deviation (SD)

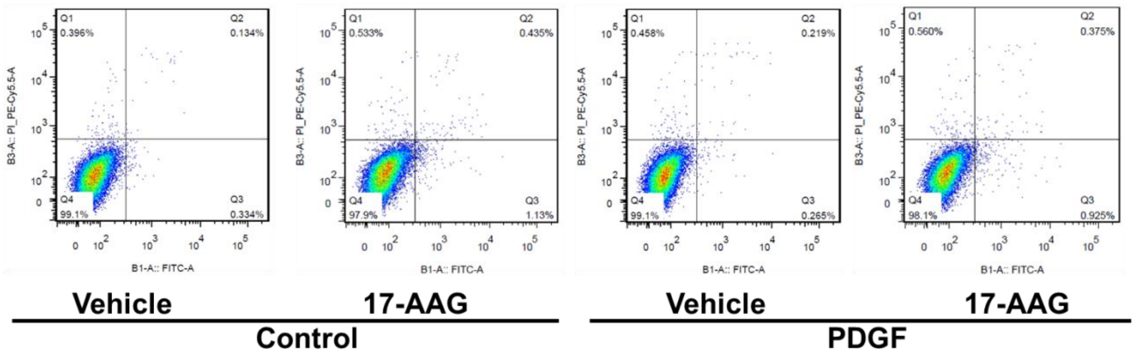


Figure S2: The affect of 17-AAG on PASMCs growth and apoptosis. PASMCs were harvested and stained by Annexin V/propidium iodide. Flow cytometric results showed 17-AAG did not induce any significant differences on apoptosis. The experiment was repeated three times with independent cells.

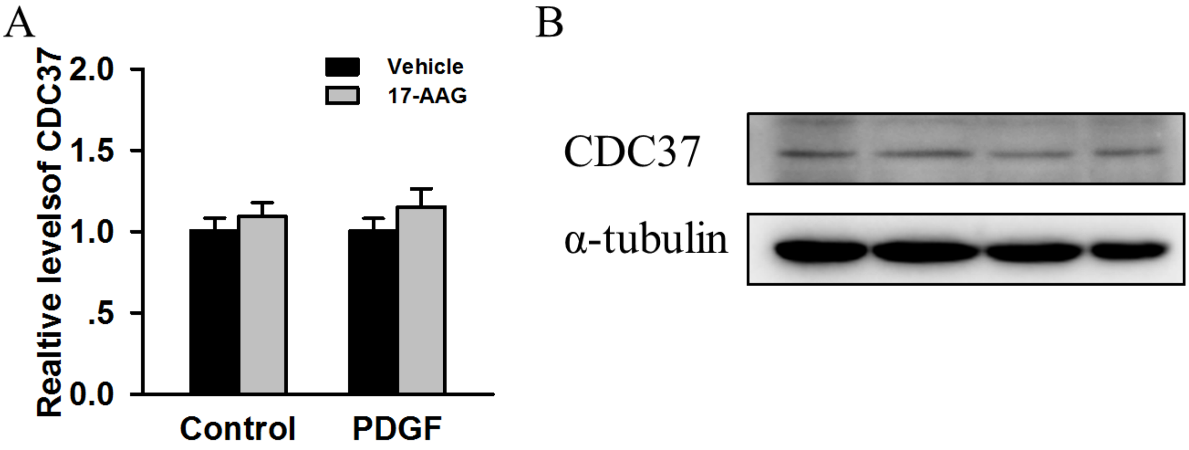


Figure S3: Affect of 17-AAG on expression of CDC37 in PASMCs with or without PDGF-bb stimulation. A, Real-time PCR analysis of CDC37 in PASMCs after 17-AAG treatment. Data represent the mean of three independent experiments ± SD. B, Western blot analysis of CDC37 in PASMCs after 17-AAG treatment. The experiment was repeated three times with independent protein samples.

Table S1: Correlation between HSP90 and clinical parameters of CHD patients with PAH

| | | WBC | NEUT | IL6 | CRP | BNP | mPAP |
|---|---|---|---|---|---|---|---|
| HSP90 | Correction Coefficient | 0.016 | -0.082 | 0.241 | 0.587 | 0.232 | 0.444 |
| | *P* | 0.935 | 0.686 | 0.225 | 0.001** | 0.244 | 0.020* |

WBC, white blood cell; NEUT, neutrophil; IL6, interleukin-6; CRP, C reaction protein; BNP, pro-brain natriuretic peptide; mPAP, mean pulmonary artery pressure. **P*<0.05 and ***P*<0.01.

Table S2: The parameter of the pulmonary arterioles determined by Image J software

| | Control (n=30) | MCT (n=6) | 17-AAG (n=11) |
|---|---|---|---|
| vessel internal diameter [μm] | 71.5±14.1 | 56.4±13.2 | 63.8±11.2 |
| vessel external diameter [μm] | 80.2±15.4 | 90.2±9.5 | 90.3±9.0 |
| vessel area (TA) [$\mu m^2$] | 4154.3±1550.9 | 2625.9±1185.3 | 3283.2±1130.7 |
| lumen area (IA) [$\mu m^2$] | 5219.2±1913.1 | 6451.4±1355.6 | 6453.7±1314.1 |

Ten pulmonary arterioles were randomly examined from hematoxylin-eosin staining images of lung tissues lesions, and were examined for structural integrity using Image J software. Data were expressed as mean ± SD.

Table S3: Quantitative analysis of immunohistochemistry staining in pulmonary arterioles

| | NF-κB p65 | | p-NF-κB p65 | | HSP70 | |
|---|---|---|---|---|---|---|
| | MCT (n=6) | 17-AAG (n=11) | MCT (n=6) | 17-AAG (n=11) | MCT (n=6) | 17-AAG (n=11) |
| immunointensity | 2.42±0.51 | 1.82±0.59(*) | 2.66±0.49 | 2.05±0.49(*) | 2.75±0.45 | 1.95±0.49(*) |
| % positive cells | 48.38±6.22 | 18.24±3.84(**) | 44.03±6.17 | 17.62±2.42(**) | 40.58±4.75 | 19.61±3.59(**) |

The protein expression was estimated by immunostaining intensity and percentage of positive cells in pulmonary arterioles. Immunostaining intensity was rated as follows: 0, none; 1, weak; 2, moderate; and 3, intense. Percentage of positive cells was calculated by positive pulmonary arterioles cells. The quantitative data were expressed as mean ± standard deviation (SD).

*$P$<0.05 and **$P$<0.01 vs MCT group.

Table S4: Primer sequences for genes detection by real-time PCR

| Gene full name | Gene Symbol | Sequence (5’→3’) |
|---|---|---|
| Cyclin D1 | CCND1 | Forwards：GCACAACGCACTTTCTTTCC<br>Reverse： TCCAGAAGGGCTTCAATCTG |
| Cyclin-dependent kinase 4 | CDK4 | Forwards：ATCTGGAGCGCAGTTGCT<br>Reverse：CCTTGTGCAGGTAAGAGTGCT |
| Cell division cycle 37 | CDC37 | Forwards：GGAGTTGGCCAAGAGTCTGA<br>Reverse： CCCTCCATGTACTGCTGGTC |
| Glyceraldehyde-3-phosphate dehydrogenase | GAPDH | Forwards：CAAGTTCAACGGCACAGTCA<br>Reverse： CCCCATTTGATGTTAGCGGG |
